# An in-silico study examining the induction of apoptosis by Cryptotanshinone in metastatic melanoma cell lines

**DOI:** 10.1186/s12885-018-4756-0

**Published:** 2018-08-29

**Authors:** Radhika S. Saraf, Aniruddha Datta, Chao Sima, Jianping Hua, Rosana Lopes, Michael Bittner

**Affiliations:** 1 Department of Electrical and Computer Engineering, Texas A&M University, College Station, US; 2 TEES-AgriLife Center for Bioinformatics and Genomic Systems Engineering (CBGSE), College Station, US; 3 Translational Genomics Research Institute (TGen), Phoenix, US

**Keywords:** Melanoma, Trail, Cryptotanshinone, Stat3, Boolean networks

## Abstract

**Background:**

Metastatic melanoma is an aggressive form of skin cancer that evades various anti-cancer treatments including surgery, radio-,immuno- and chemo-therapy. TRAIL-induced apoptosis is a desirable method to treat melanoma since, unlike other treatments, it does not harm non-cancerous cells. The pro-inflammatory response to melanoma by nF *κ*B and STAT3 pathways makes the cancer cells resist TRAIL-induced apoptosis. We show that due to to its dual action on DR5, a death receptor for TRAIL and on STAT3, Cryptotanshinone can be used to increase sensitivity to TRAIL.

**Methods:**

The development of chemoresistance and invasive properties in melanoma cells involves several biological pathways. The key components of these pathways are represented as a Boolean network with multiple inputs and multiple outputs.

**Results:**

The possible mutations in genes that can lead to cancer are captured by faults in the combinatorial circuit and the model is used to theoretically predict the effectiveness of Cryptotanshinone for inducing apoptosis in melanoma cell lines. This prediction is experimentally validated by showing that Cryptotanshinone can cause enhanced cell death in A375 melanoma cells.

**Conclusion:**

The results presented in this paper facilitate a better understanding of melanoma drug resistance. Furthermore, this framework can be used to detect additional drug intervention points in the pathway that could amplify the action of Cryptotanshinone.

**Electronic supplementary material:**

The online version of this article (10.1186/s12885-018-4756-0) contains supplementary material, which is available to authorized users.

## Background

Melanoma is one of the most prevalent and aggressive forms of skin cancer. Normal melanocytes are the light receptors in the skin and are equipped to protect and repair the body from damage caused by radiation. The chemoresistance of melanoma cell lines has been attributed to their inherent capability to survive. In melanoma cells in particular, and cancer cells in general, this survival mechanism is hijacked by the mutated genes and exploited to counter medical treatment [1].

The human body reacts to threats by relying on its immune system and by appropriate functioning of the cellular signaling pathways. TNF-related apoptosis-inducing ligand (TRAIL) is implicated in immunosurveillance, which is the ability of the immune system to recognize pathogens and activate the mechanisms to neutralize their effect [2]. TRAIL resistance is observed in melanoma cell lines; it is associated with the mutations in cell survival pathways [3, 4].

Abnormalities in cell cycle control are a characteristic of cancer, and this is accompanied by uncontrolled growth [5]. Drugs used to treat melanoma try to restore the normal cell cycle function through action on the cell survival pathways. Metastatic melanoma cells are known to develop resistance to most of the commonly used drugs and therapy [1]. Chemoresistance is linked with TRAIL resistance in melanoma [4]. Treatment strategies that involve sensitization of the melanoma cells to TRAIL-induced apoptosis have shown promise [6]. Cryptotanshinone is one of the drugs that has been shown to restore TRAIL sensitivity [7].

**Fig. 1:**
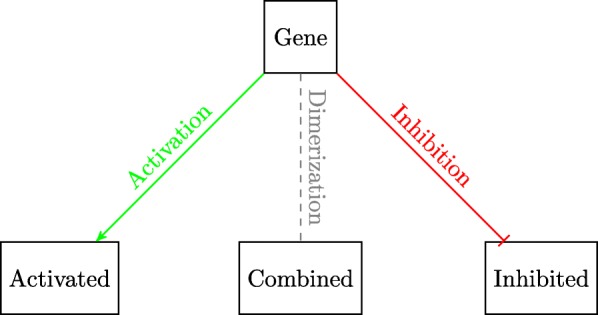
Color coding for gene interactions in Figs. 3 through 8

**Fig. 2:**
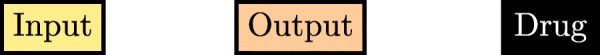
Legend for use in Figs. 3 through 8

**Fig. 3:**
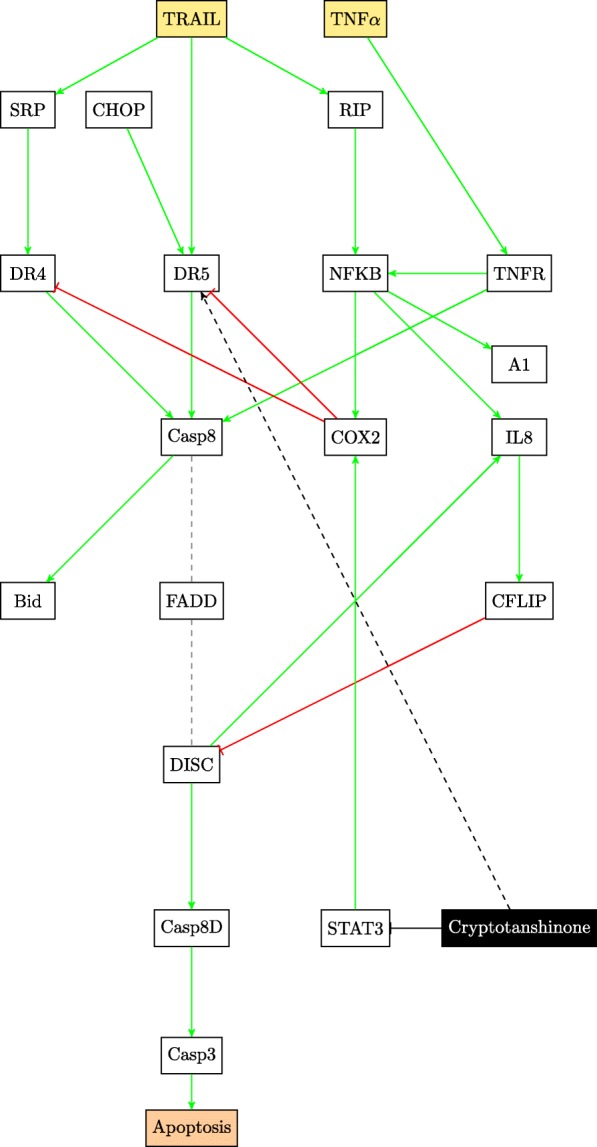
Extrinsic Apoptosis and the nF *κ*B pathways

This paper will model the development of drug resistance in metastatic melanoma cells, using a Boolean network to explain the induction of apoptosis by Cryptotanshinone. The paper is organized as follows. The first section describes the functions of the various pathways in cancer and how they contribute to drug resistance. The following section describes the Boolean formalization of these pathways. Finally, the theoretical results are presented, followed by the experimental validation in the last section. For clarity of presentation, the color schemes shown in Figs. 1 and 2 will be used while schematically modeling signaling pathways and the interactions between genes. Extensive use of these schemes can be seen in Figs. 3 through 8 to follow.

### Biological pathways in melanoma

The various gene interactions in melanoma can be represented by biological pathways, which are all well documented [8–10]. Some of the interconnections derived during modelling these pathways are based on the interpretation of different research papers [3, 11–22] by the authors of the present paper. We consider only a subset of all possible interconnections and signaling pathways in the cell, since the cancer of interest to us here is melanoma.

TRAIL resistance is attributed to the activation of the nF *κ*B pathway and the cell survival pathways. Pro-inflammatory response of nF *κ*B leads to the overexpression of cFLIP (Cellular FLICE (FADD-like IL-1 *β*-converting enzyme)-inhibitory protein) that interferes with the formation of the death-inducing signaling complex (DISC), an important step in the extrinsic apoptosis governed by TRAIL [3, 23]. This is clearly shown in Fig. 3.

Another possible reason for the development of TRAIL resistance is due to the lower expression of death receptors - death receptor 4 (DR4) and 5 (DR5) [4]. TRAIL receptors are abundantly expressed in the early stages of melanoma, however as the immune system fails to combat cancer growth, the TRAIL-induced apoptosis is affected.

The cell survival pathways mTOR/PI3K/AKT and MAPK/ERK have been implicated as contributors to TRAIL resistance [4, 24]. These two anti-apoptotic pathways govern melanocytes; control their cell cycle, promote proliferation, growth and survival [14]. They may be involved in melanomagenesis, particularly N-Ras, B-Raf and PTEN loss are some of the commonly occurring mutations of Ras, Raf and PTEN respectively [8–10]. These pathway mutations can attenuate the cytotoxicity of several drugs [24].

**Fig. 4:**
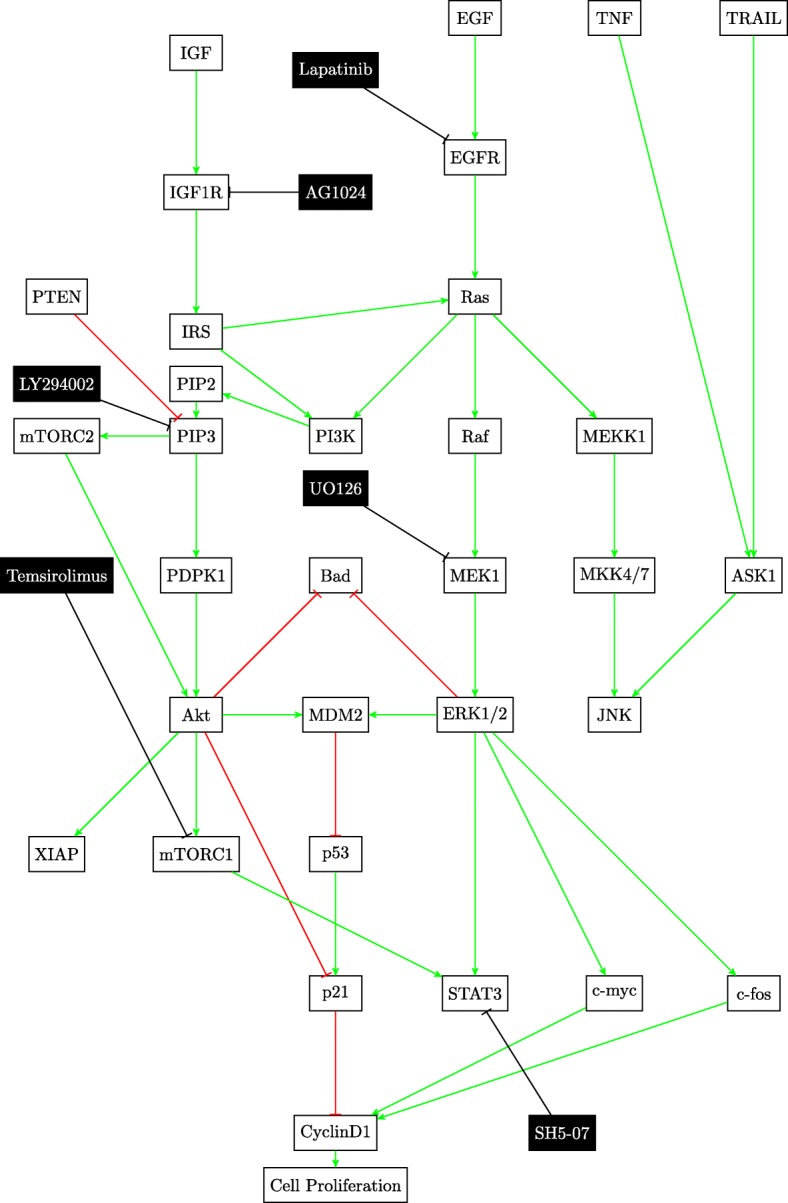
JNK, p53, PI3K/AKT/mTOR and MAPK/ERK pathways

**Fig. 5:**
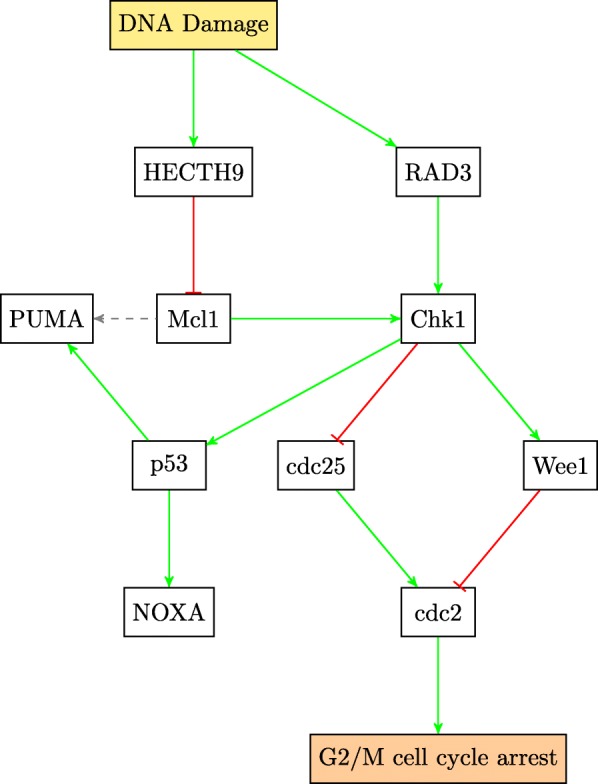
DNA damage pathway

Figure 4 shows the crosstalk between the two cell survival pathways and how they mutually control the p53 pathway. The tumor suppressor gene p53 is also considered to be an oncogene. Referred to as the master guardian gene, p53 responds rapidly to DNA damage [25]. Figure 5 shows how the cell cycle arrest can occur if DNA damage is detected and can lead to the activation of the tumor suppressor action of p53 [26]. Once activated, p53 serves as a brake on cell proliferation as shown in Fig. 4.

**Fig. 6:**
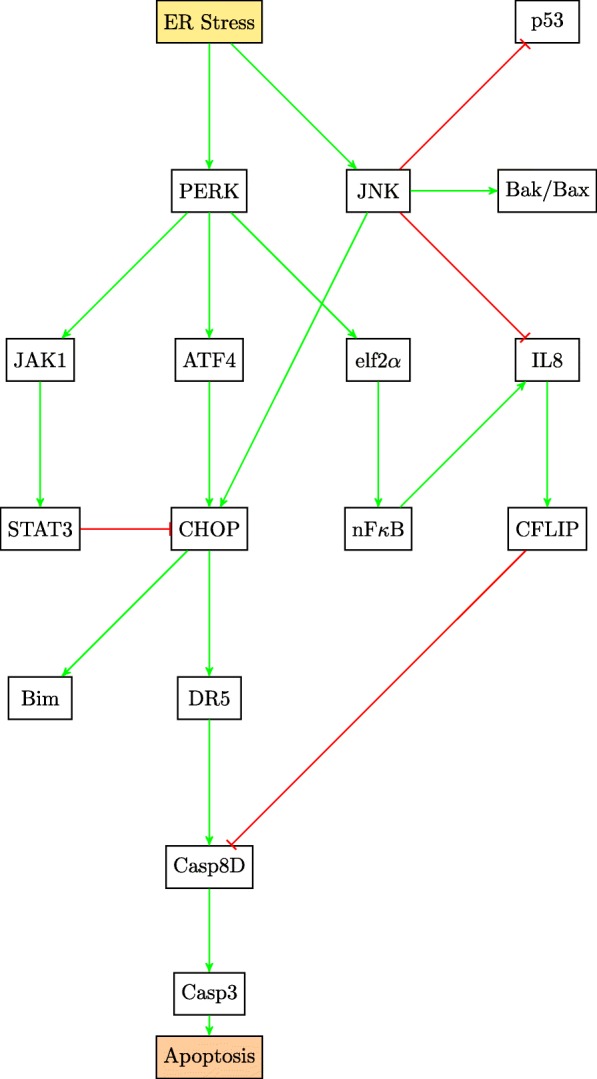
Endoplasmic Reticulum Stress and the JNK pathway

There are other pathways which are involved in TRAIL resistance indirectly, such as the pathway governing the unfolded protein response (UPR). UPR is triggered by endoplasmic reticulum (ER) stress as depicted in Fig. 6. In melanoma, UPR may aid metastasis via the epithelial-mesenchymal transition (EMT)[27]. UPR could be linked to chemoresistance and TRAIL resistance, as it activates the pro-inflammatory response. JNK is also activated in response to ER stress, it inhibits IL8 signaling and increases TRAIL-induced apoptosis [28]. JNK is also involved in the upregulation of CHOP and Bak/Bax, both of which are pro-apoptotic factors [29]. These relationships involving JNK are captured in the pathway diagram in Fig. 6.

Signal transducer and activator of transcription 3 (STAT3) plays a part in decreasing TRAIL cytotoxicity in metastatic melanoma cells. Cyclooxygenase-2 (COX2) is a transcriptional target of both nF *κ*B and STAT3, and is a regulator of inflammatory response. Inhibition of STAT3 causes a decrease in protein expression of COX2 [30]. STAT3 is also activated upon incidence of ER stress by PERK [31]. The increase of metastatic activity by UPR is partly due to the action of STAT3 [31]. Additionally, STAT3 upregulates Mcl1, an anti-apoptotic factor, thus contributing to cell survival [17].

**Fig. 7:**
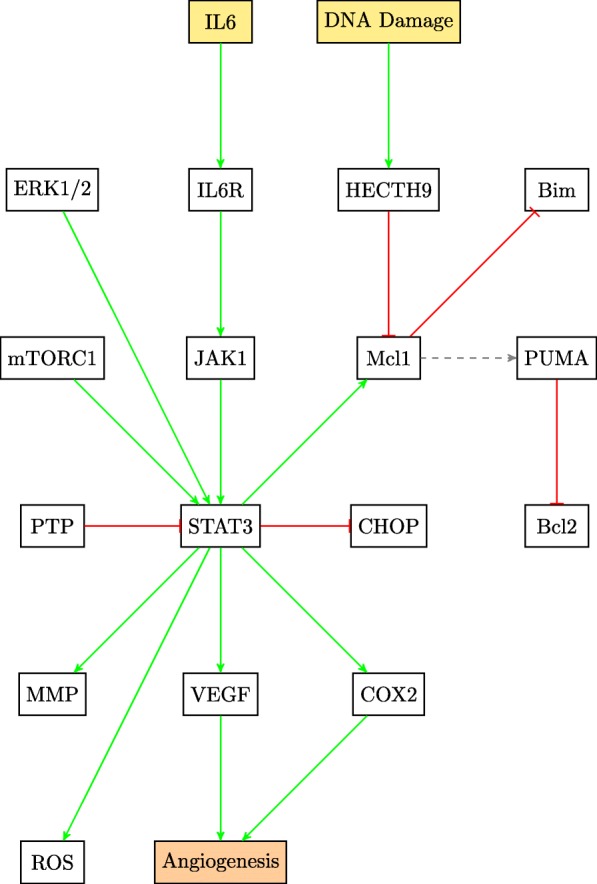
STAT3 pathway

The role of STAT3 in cancer cells is extensive as is evident from the pathway diagram in Fig. 7. STAT3 is activated in the skin to achieve migration of keratinocytes, that produce proinflammatory mediators and initiate immune response [32]. It regulates reactive oxygen species (ROS) in the mitochondria. ROS levels influence mitochondrial membrane potential and are important driving factors in mitochondrial apoptosis and are shown to have an effect on TRAIL sensitivity [33–35]. Given its influence on the various pathways involved in developing TRAIL resistance, STAT3 is a good candidate to induce TRAIL sensitivity [36, 37].

**Fig. 8:**
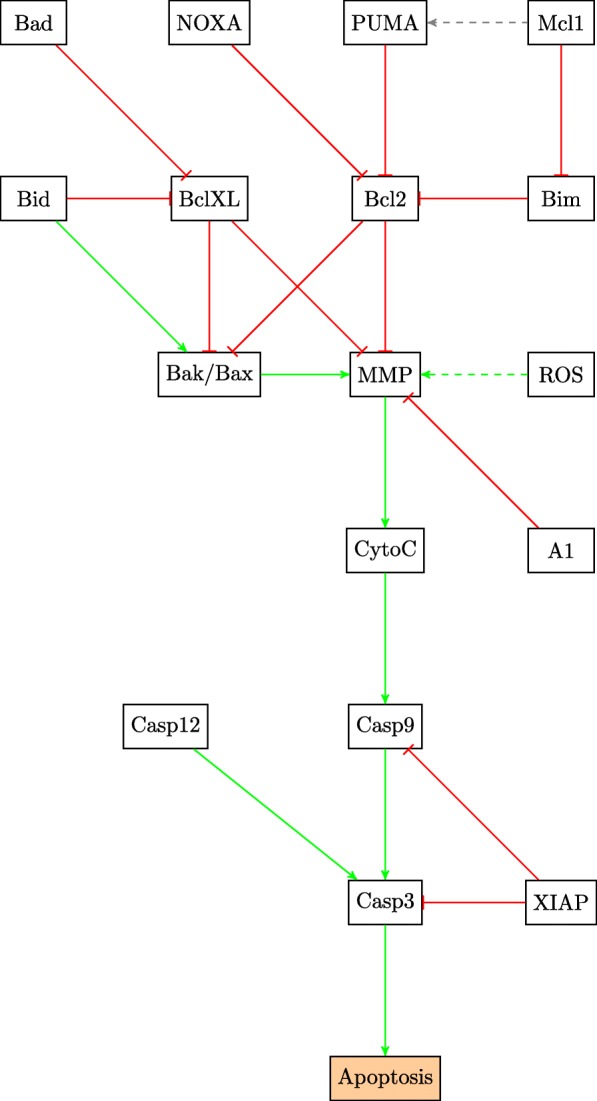
Mitochondrial Apoptosis Pathway

There are several existing drugs that act at different points in the MAPK/ERK and mTOR/PI3K/Akt pathways as is shown in Fig. 4; however none of them have been proven significantly effective against melanoma [1]. A possible mechanism for drug resistance is the failure to induce apoptosis in cancer cells. Typically, most cancer cells deactivate the pathways to apoptosis and simultaneously heighten the activities of the cell proliferation and growth pathways [5]. The balance of pro-apoptotic and anti-apoptotic factors determines the fate of the cell [12, 13]. These factors are regulated by genes in different signaling pathways as can be seen in Table 1. The mitochondrial pathway which governs cellular respiration and apoptosis in many cells is shown in Fig. 8. The matrix membrane permeability depends on the ratio of the pro-apoptotic to the anti-apoptotic factors and is controlled by the matrix metalloproteases (MMPs) [13]. It is noteworthy that in both normal and cancer cells, the expression of pro-apoptotic factors can be detected [3]. This indicates that the upstream defects in cancer most likely inhibit apoptosis by an increase in the activity of anti-apoptotic genes. This fact is useful when trying to understand drug resistance.

**Table 1:** Mitochondrial apoptosis factors

Factor	From pathway	Effect on apoptosis
Bad	AKT	Pro-apoptotic
Bid	TRAIL	Pro-apoptotic
Casp-12	ER stress	Pro-apoptotic
Noxa	p53	Pro-apoptotic
Puma	p53	Pro-apoptotic
Bim	JNK	Pro-apoptotic
Mcl-1	STAT3 and DNA damage	Anti-apoptotic
A1	nF *κ*B	Anti-apoptotic
Bak/Bax	Mitochondrial	Pro-apoptotic
Bcl2	Mitochondrial	Anti-apoptotic
Bcl-XL	Mitochondrial	Anti-apoptotic
XIAP	AKT	Anti-apoptotic
ROS	STAT3, TRAIL, TNF *α* and ER stress	Pro-apoptotic

### Cryptotanshinone as an effective drug

Cryptotanshinone (CT) is one of the bio-active compounds of the plant *Salvia miltiorrhiza* (danshen), the root extract of which has been used widely in traditional Chinese herbal treatment for various diseases. There are many studies discussing the effects of CT on cancer [38–40], and on melanoma [7, 18, 30, 41]. Cryptotanshinone has been shown to kill tumor-initiating cells (cancer stem cells) by targeting stemness genes [40], cause cell cycle G0/G1 and G2/M phase arrest, counter metastasis and invasion of cancer cells [18], and activate the mitochondrial [41] as well as the extrinsic apoptotic pathways [7, 30]. Its protein structure and molecular targets have been studied in efforts to make it an effective drug for cardiovascular disease [38], and even for cancer [42].

CT can restore TRAIL sensitivity and induce apoptosis in A375 melanoma cells, by increasing DR5 expression via the induction of CHOP (CCAAT/enhancer-binding protein-homologous protein) [7]. In addition, STAT3 plays a key role in and is upstream of many of the functions that CT affects and is a known target of CT in other cancers [43, 44].

## Methods

We model the biological signaling pathways that we have discussed in the “Background” section as a Boolean network. Each gene is a node and its direct interaction with another gene is represented as an edge. Gene expression is binarily quantized: a gene, if expressed is considered to be ON (State 1) and if not expressed, is considered to be OFF (State 0). If two or more genes interact to activate or inhibit a third gene, such relationships are modelled with the use of logic gates. The genetic regulatory network can then be thought of as a multi-input multi-output (MIMO) digital logic circuit.

**Fig. 9:**
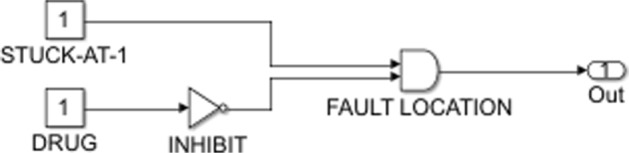
Boolean representation of the drug action countering a stuck-at-one fault

**Fig. 10:**
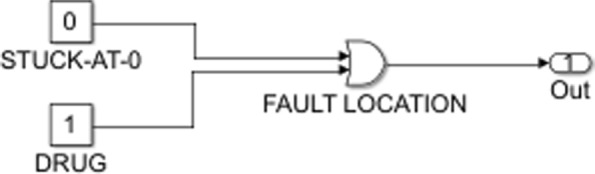
Boolean representation of the drug action countering a stuck-at-zero fault

A cancerous cell will not have the same input-output mapping as a normal one. This is due to the abnormalities that occur in the biological pathways of cancer cells. Malfunctioning genes lead to uncontrolled cell proliferation, increased inflammation and failure of the apoptotic pathways. These irregularities of tumor cells can be thought of as faults in the Boolean network, particularly stuck-at faults. A stuck-at fault occurs when a node in the network is permanently set to a fixed value of either zero (stuck-at-0 fault) or one (stuck-at-1 fault) [5]. This implies that the circuit will not change as expected when subjected to a certain set of inputs. The output vector of a faulty network then will be independent of the other signal values in the regulatory circuit. An over-expressed gene can be denoted as a stuck-at-1 fault. This notion is common in cancer where oncogenes tend to display similar faulty behaviour, irrespective of what input they receive and evade any corrective action from upstream. The effect of such a fault can be corrected by using a drug as shown in Fig. 9. On the other hand, a stuck-at-0 fault can result when a gene becomes permanently inactive, independent of the activity status of its upstream regulators. For example, a mutated p53 gene in a cancer cell will remain inactive despite being phosphorylated as a result of cellular DNA damage. This situation, common to several cancers, is one where a drug can correct a stuck-at-0 fault as shown in Fig. 10. The static Boolean network considered here is used to represent a trail resistant network and also includes information about how drug intervention could allow us to sensitize the melanoma cell lines to TRAIL. We focus on the TRAIL apoptotic pathway and on the effect the genes in the other pathways have on extrinsic cell death. The other inputs are DNA damage, ER stress and the growth factors that activate the pathways involved in melanoma. The outputs are all apoptotic factors, both pro- and anti- apoptotic, the ratio of which will decide whether the cell undergoes death. The input and output vectors are given by Eqs. 1 and 2 below:

1 $$ {\begin{aligned} \text{Input}=\ & [\!\text{ER Stress, TNF }\alpha, \text{TRAIL, PTP, IL6,}\\ &\text{DNA Damage, IGF, EGF}] \end{aligned}}  $$

2 $$ {\begin{aligned} \text{Output} =\ & [\text{Casp8, Bid, Bad, Bim, Bak/Bax, Casp12,}\\ &\text{Bcl-XL, Bcl2, XIAP, Mcl1}] \end{aligned}}  $$

For A375 melanoma cells, we consider 6 possible faults in our model. These correspond to the common mutations in the involved pathways and especially those that have been shown to cause TRAIL resistance [24]. All possible combinations of the faults have been simulated, that is 64 different configurations of the fault vector are considered. It is important to note that each component of the fault vector is either zero or one based on whether a particular fault is present or not. A one in the fault vector can denote a stuck-at-one fault or a stuck-at-zero fault, whichever is consequential for that particular gene. For instance, if the fault vector is [1 0 0 0 0 0], this implies that the Ras gene is faulty. Since it is a stuck-at-one type of fault, it means that Ras is being constitutively expressed. On the other hand, presence of a stuck-at-zero fault represents the downregulation of the gene. For instance, when the fault vector equals [0 0 1 0 0 0], it means that PTEN is faulty and its suppressing action has failed. The fault vector components are given by Eq. 3 and the types of faults are as listed in Table 2.

3 $$ \text{Fault} =\ [\!\text{Ras, Raf, PTEN, p53, STAT3, DR5}]  $$

**Table 2:** Faults

Stuck at 1	Stuck at 0
Ras	PTEN
Raf	p53
STAT3	DR5

The activity points of the different drugs on the pathways have already been shown in Figs. 3 and 4. The components of the drug vector are displayed in Eq. 4.

4 $$ {\begin{aligned} \text{Drugs} =\ & [\!\text{CT, LY294002, Temsirolimus, UO126,} \\ &\text{Lapatinib, SH5-07, AG1024}] \end{aligned}}  $$

Each component of the drug vector corresponds to whether or not that drug is applied, so a zero in the *i*^*th*^ column indicates that the *i*^*th*^ drug is not applied and vice versa. Since a major goal of this paper is to evaluate the action of Cyrptotanshinone, either by itself, or for enhancing the activity of other drug combinations, the combination of drugs considered here is limited to Cryptotanshinone alone and Cryptotanshinone in combination with the other drugs. Since there are six other drugs in the vector, a total of 2^6^ drug combinations were tested. For instance, the drug vector [1 0 0 0 0 0 0] indicates that only Cryptotanshinone is applied.

**Fig. 11:**
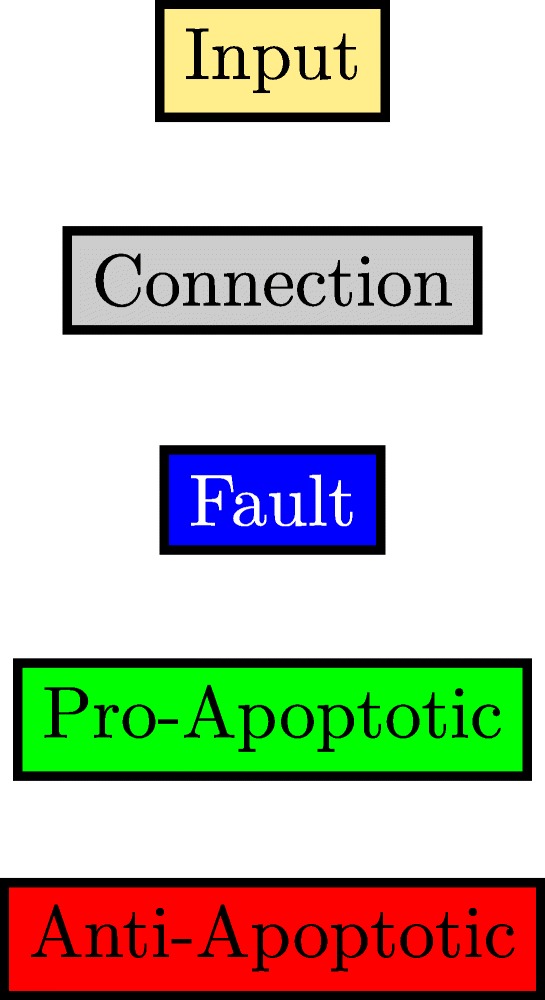
Legend showing the color coding scheme used in Figs. 12, 13 and 14

**Fig. 12:**
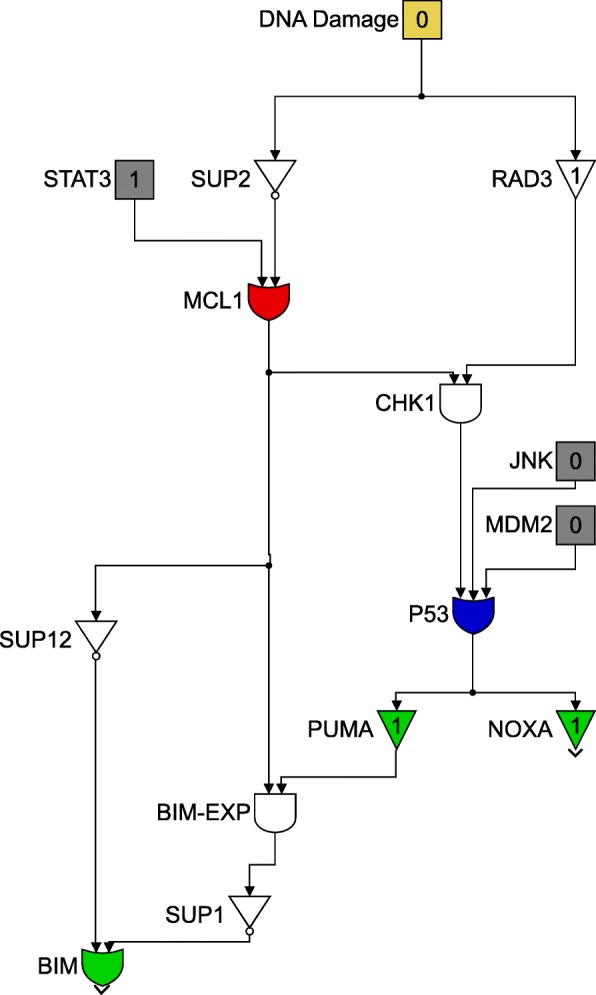
Boolean network for the DNA Damage pathway

**Fig. 13:**
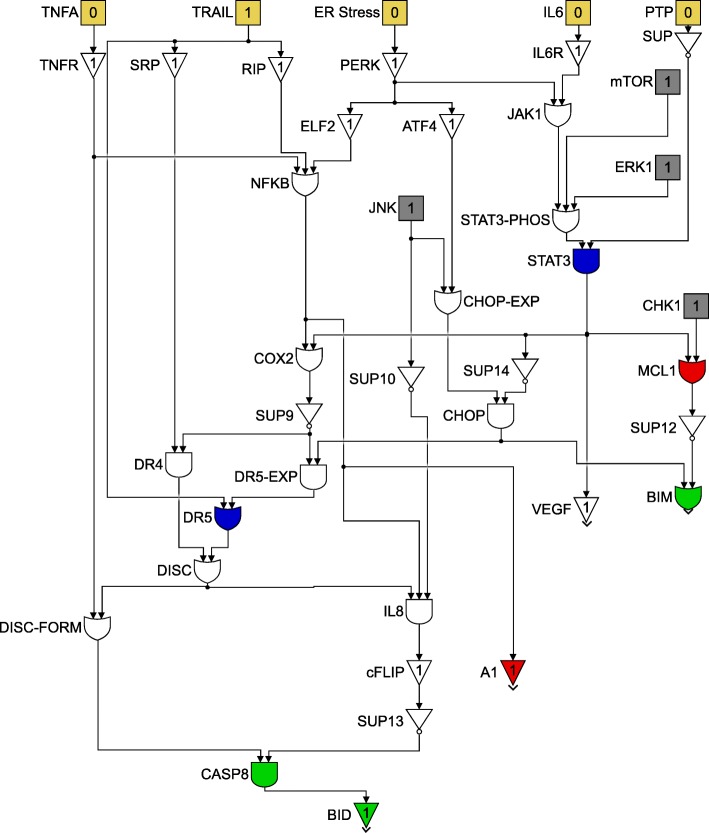
Boolean network for the TRAIL, ER Stress and STAT3 pathway

**Fig. 14:**
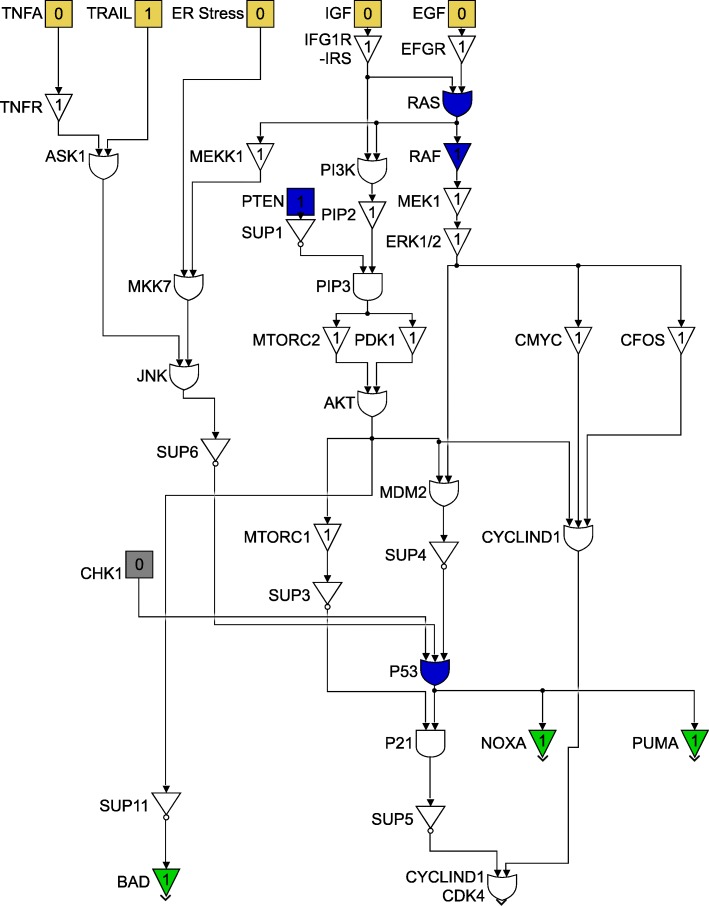
Boolean network for the PI3K/AKT/mTOR and MAPK/ERK pathway

For clarity of exposition, the entire Boolean network will be split up into three different components. Each component will follow the colour scheme shown in Fig. 11 and the interconnections between the three component networks will be indicated by the gray blocks. The three components are shown in Figs. 12, 13 and 14. Figure 12 shows the relationship between the DNA damage input and how the apoptotic factors are affected upon the incidence of DNA damage, and this figure also helps in closely studying the effect of a p53 fault. Similarly, Figs. 13 and 14 represent the gene interactions in the major pathways involved in melanoma. An additional Simulink file shows the entire Boolean network as a whole [see Additional file 1].

## Results and discussion

We ran several rounds of simulations to test how Cryptotanshinone acts in combinations with the other drugs. To check the effectiveness of CT in increasing TRAIL cytotoxicity, we monitor its influence on the apoptosis induced. In this section, we are testing a TRAIL resistant static Boolean network. Here, it should be pointed out that a network can display trail resistance even in the absence of TRAIL, the resistance in that case having been residually left over from an earlier TRAIL induction event. The metric used to calculate the degree of apoptosis is:

$$\text{Apoptosis Ratio} = \frac{\sum \text{Pro-Apoptotic factors}}{\sum \text{Anti-Apoptotic factors}} $$

The apoptosis ratio is a measure of the relative change in apoptosis upon a change in conditions. The apoptosis ratio will change depending on different factors such as the values of the inputs, the presence of certain faults or the application of a drug. Changing the input combination to the Boolean network will change the value of the apoptosis ratio. Figure 15 presents three different states of the Boolean network, when the input vectors are:

1. ‘0000000’ : ‘No Input’ which means that no growth factors, cytokines or stress signals are present and the STAT3 suppressor PTP is OFF.

**Fig. 15:**
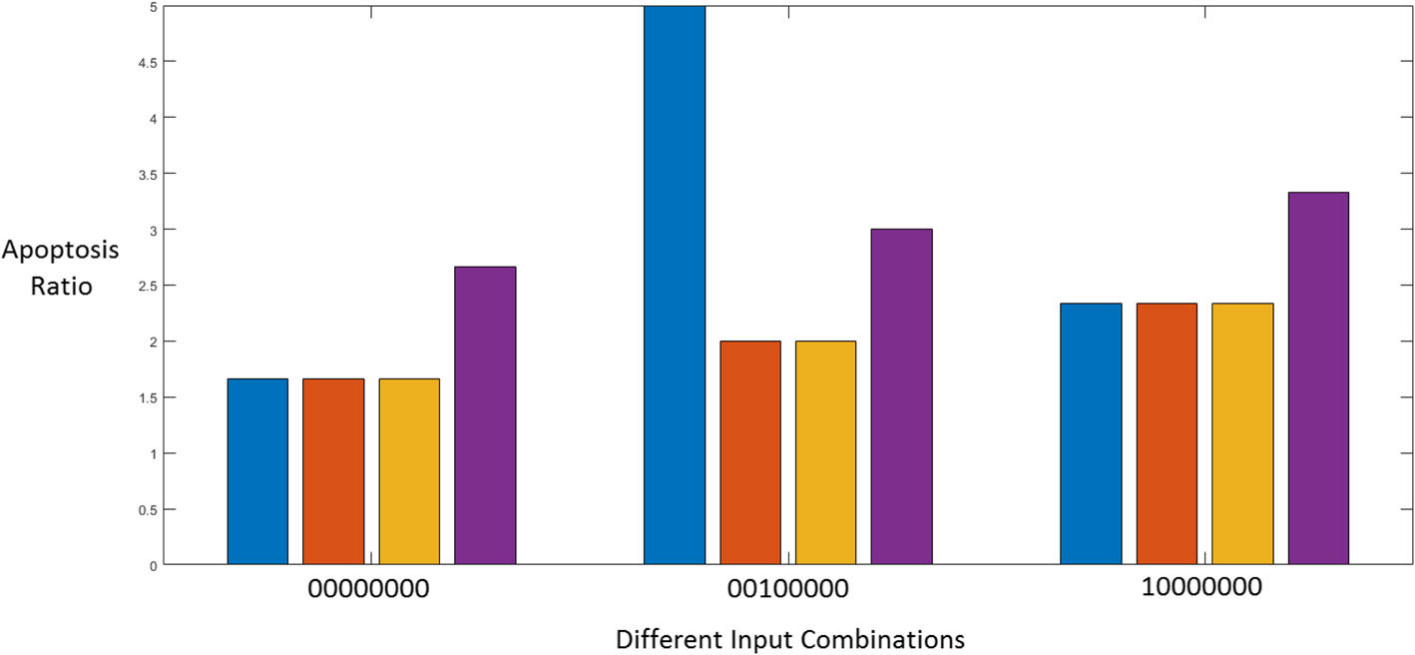
Apoptosis ratios for different inputs
2. ‘0010000’ : ‘TRAIL-induced apoptosis’ which means that the TRAIL apoptotic pathway is active.
3. ‘1000000’ : ‘ER Stress induced Apoptosis’ which considers ER Stress as the only active input.

Each color in the figure represents a different fault and drug combination. Blue stands for the situation where there is no fault and no drug; orange means that the DR5 and STAT3 faults are present; yellow shows the apoptosis induced by SH5-07 in the presence of these faults; and violet shows the apoptosis induced by CT in the presence of the two faults.

From Fig. 15, we can see that the apoptosis ratio is 1.67 when there is ‘No Input’ and ‘No Fault’. Moreover, we observe that CT is inducing apoptosis even in the absence of TRAIL or other apoptosis-inducing factors. This means that CT must be down-regulating the anti-apoptotic factors through its action on STAT3, thus leading to a relatively greater value of the apoptosis ratio.

A similar situation can be seen for the ‘ER Stress induced apoptosis’ case, where the apoptosis value increases upon application of CT. However, only its effect on STAT3 is not enough to explain the increased TRAIL sensitivity. This is clear by looking at the action of the other STAT3 inhibitor SH5-07, which is unsuccessful in inducing further apoptosis in the presence of the faults. Here, it is evident that the upregulation of DR5 by CT plays a role in increasing the apoptosis ratio.

Looking at the ‘TRAIL-induced apoptosis’ condition in the absence of a fault, we observe that the apoptosis ratio is large. DR5 and STAT3 faults reduce the value to almost half. The STAT3 inhibitor SH5-07 is unable to counter these faults. Cryptotanshinone though not able to regain the fault-free value of apoptosis, is effective in increasing apoptosis despite the presence of faults. This seems to imply that the upregulation of DR5 is instrumental to restoring TRAIL sensitivity.

**Fig. 16:**
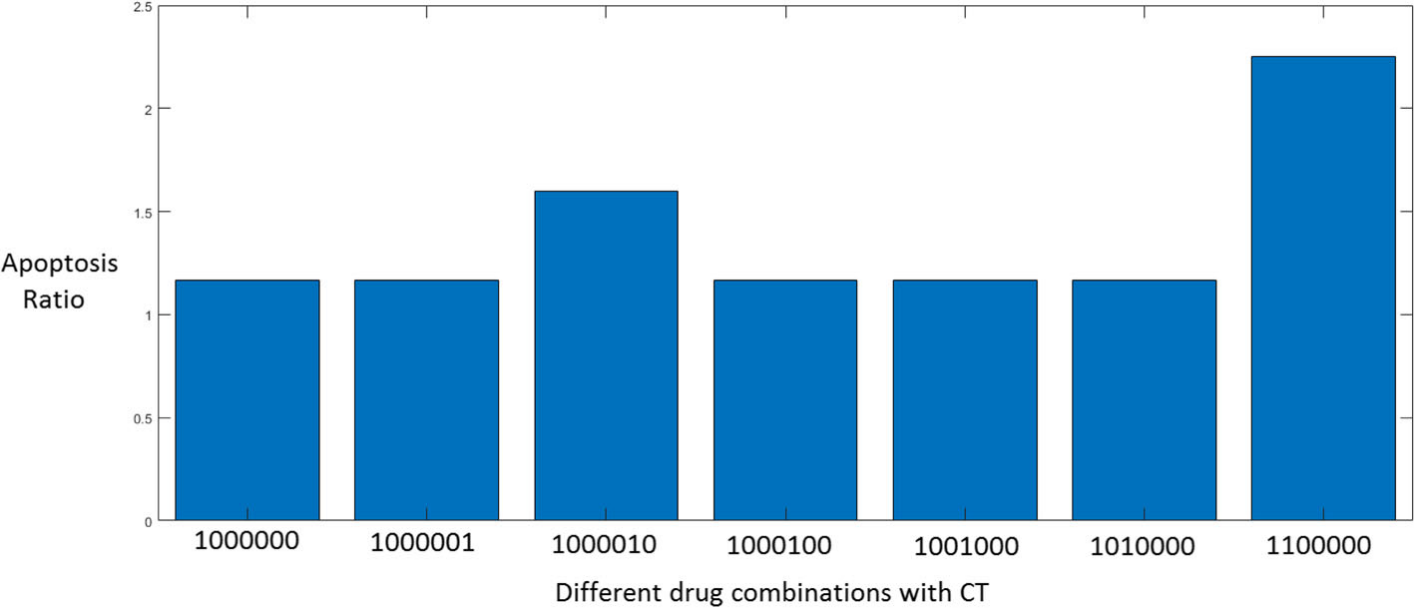
Apoptosis by CT in combination with a single drug in the presence of simultaneous occurrence of all faults

The next simulation was run to test which single drug is the most effective in combination with CT. We considered the input to be TRAIL so that the input vector is ‘0010000’ and assumed that all 6 faults are simultaneously present. The results are shown in Fig. 16. The effect of LY294002, a PI3K inhibitor in combination with Cryptotanshinone seems to be better than the other combinations considered. The role of the mTOR/PI3K/AKT pathway in TRAIL resistance is confirmed by the increase in TRAIL cytotoxicity via inhibition of PI3K.

**Fig. 17:**
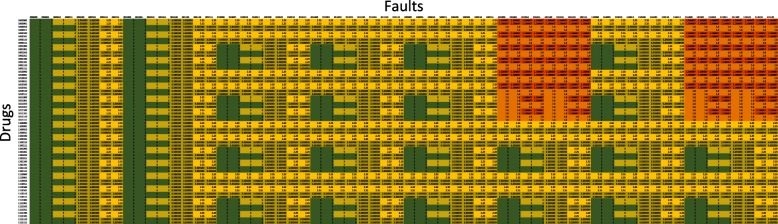
All possible combinations of faults and drugs when the input is TRAIL, with Cryptotanshinone

**Fig. 18:**
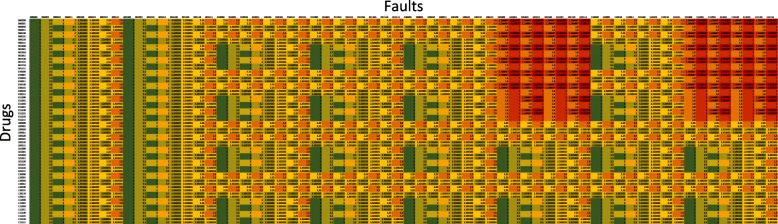
All possible combinations of faults and drugs when the input is TRAIL, without Cryptotanshinone

The final simulation evaluates all fault combinations with all the drug combinations with and without Cryptotanshinone in Figs. 17 and 18 respectively, when only the TRAIL input is active. Each row corresponds to a different drug combination (indicated by the corresponding drug vector) while each column corresponds to a different fault combination (indicated by the corresponding fault vector). The apoptosis value in each cell in the figure, thus, is the action that a drug vector has on that particular fault vector. Both the figures follow the same color scale. The red areas show regions of low apoptosis (apoptosis ratio = 0.67) while the green areas show regions of maximum apoptosis (apoptosis ratio = 5). A visual inspection shows that CT is successful in increasing TRAIL cytotoxicity for most combinations of faults. It is our conjecture in this paper that the effect of Cryptotanshinone on TRAIL resistance is through its action on STAT3 and DR5. The simulations seem to support this as they show that even in the presence of faults in other cell signaling pathways, such as p53, CT can solely through its action on STAT3 and DR5 diminish TRAIL resistance. Figure 17 does not have a single red cell, which means that CT is more effective in inducing apoptosis than any other drug combination considered in this paper. In contrast, Fig. 18 has fewer green cells, which seems to point towards Temsirolimus, an mTORC1 inhibitor [20] to perform better than the other drugs in the absence of Cryptotanshinone. LY294002 in combination with CT seems to be the most effective drug among the ones considered in this paper. This is also what was seen in Fig. 16. The red regions in Fig. 18 correspond to a PTEN fault being active and the PI3K inhibitor LY294002 seems to keep the apoptosis ratio away from the red region despite the presence of PTEN faults. This adds to the argument that the mTOR/PI3K/AKT signaling pathway contributes to TRAIL resistance, and its inhibition increases TRAIL sensitivity. An additional excel file shows the data in Figs. 17 and 18 in greater detail [see Additional file 2].

### Experimental Validation

**Fig. 19:**
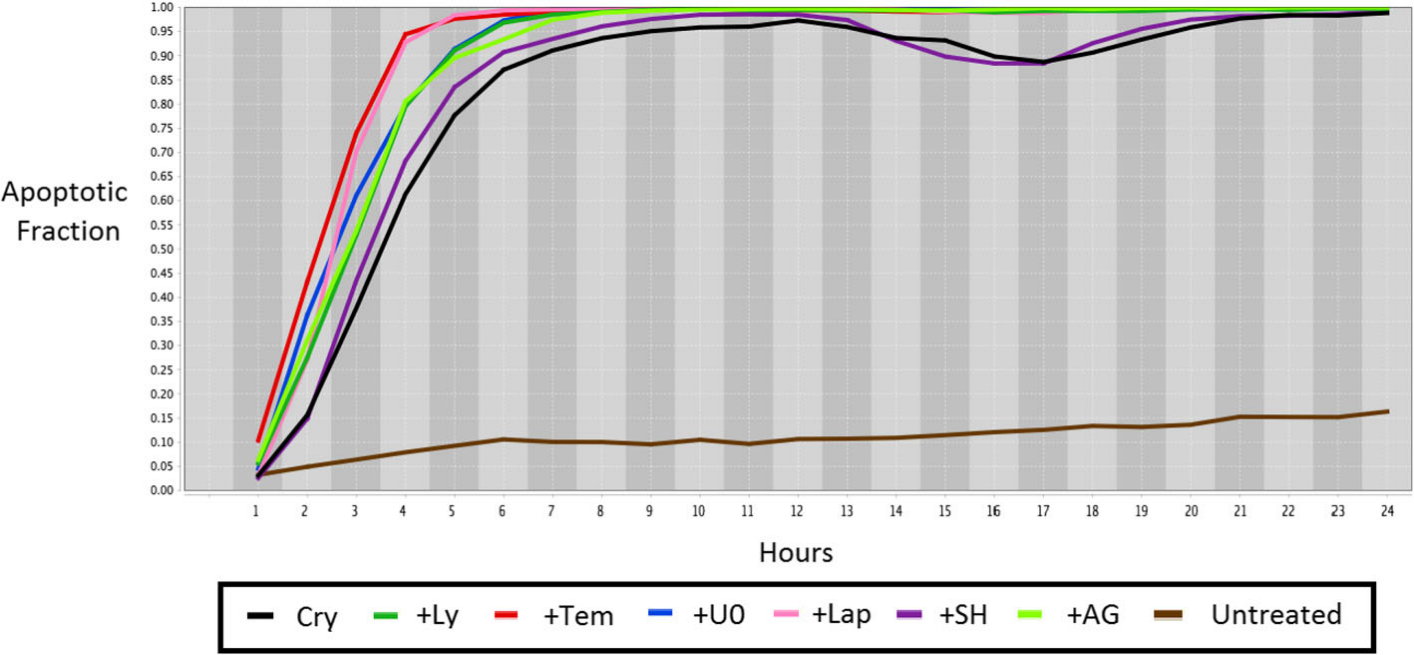
Experimental results for each single drug in combination with CT

High-content fluorescent protein reporter imaging is used to track cellular apoptosis in a sample of A375 melanoma cells, subject to various drug treatments. A two-part data processing procedure similar to the one introduced in [45] is applied to extract cell processing dynamics. The data obtained after image processing is summarized into expression profiles and represented as plots to facilitate further evaluation. The cellular apoptosis occurring in A375 melanoma cells with respect to time is displayed in Fig. 19. The Y-axis shows the apoptotic fraction, which corresponds to the percentage of apoptosis occurring in the cell line in the given time. Table 3 explains the legend in Fig. 19 in greater detail.

**Table 3:** Legend for Fig. 19

Abbreviation	Drug combination
Cry	Cryptotanshinone 50*μM*
+Ly	LY294002 10*μM*+ Cryptotanshinone 50*μM*
+Tem	Temsirolimus 10*μM*+ Cryptotanshinone 50*μM*
+U0	U0126 10*μM*+ Cryptotanshinone 50*μM*
+Lap	Lapatinib 10*μM*+ Cryptotanshinone 50*μM*
+SH	SH5-07 10*μM*+ Cryptotanshinone 50*μM*
+AG	AG1024 10*μM*+ Cryptotanshinone 50*μM*
Untreat	Untreated

It can be seen that CT in combination with the drugs one at a time is successfully inducing apoptosis in melanoma cell lines. The final value of apoptosis is similar for each combination as is also shown in Fig. 16.

## Conclusions

We modelled the TRAIL resistant metastatic melanoma network using a Boolean network. The effects of Cryptotanshinone in combination with a few other drugs were studied. Simulations were run to study the effectiveness of Cryptotanshinone in increasing TRAIL sensitivity. The theoretically predicted efficacies seem to be borne out by the experimental results.

## Additional files

Additional file 1 This is the representation of a Simulink model (.slx file). The model shown was used as a reference to cross-check the results from the.m-file. It contains the exacts same Boolean network as is encoded in ’boolean_net.m’ and is shown in the Figs. 12,13 and 14. (PDF 110 kb)

Additional file 2 This is an Excel file, it is used to analyze the data from the simulations. It has two sheets ’cry’ and ’nocry’ used to generate the Figs. 17 and 18 respectively. (XLSX 41 kb)

## Abbreviations

CT: Cryptotanshinone
MIMO: Multiple input multiple output
