# Supplementary figures and images for: An in-silico study examining the induction of apoptosis by Cryptotanshinone in metastatic melanoma cell lines
Source: BMC Cancer. 2018 Aug 29;18:855. doi: 10.1186/s12885-018-4756-0 (PMC6116360; doi:10.1186/s12885-018-4756-0)

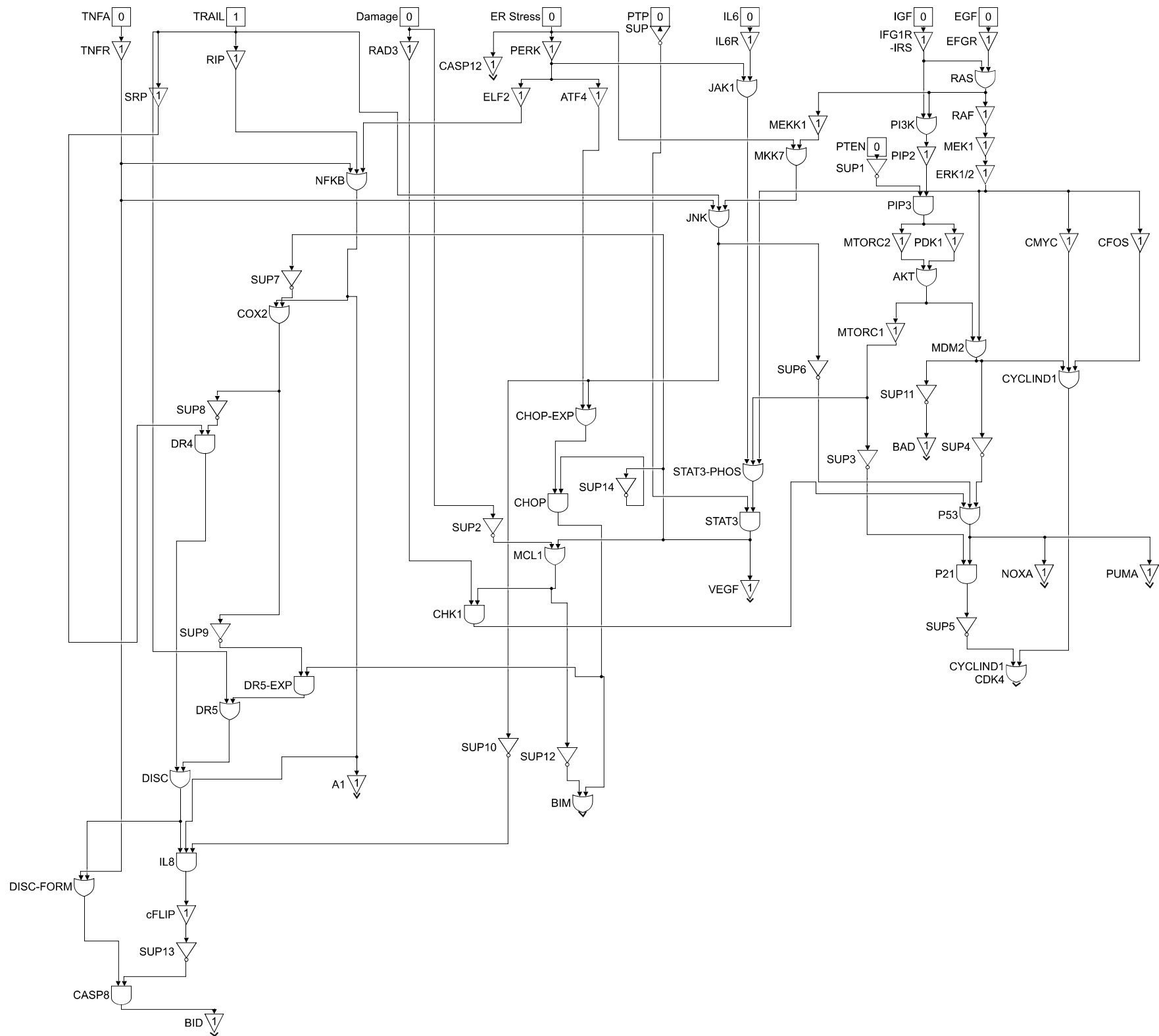

Supplement: Supplementary file 1 — This is the representation of a Simulink model (.slx file). The model shown was used as a reference to cross-check the results from the.m-file. It contains the exacts same Boolean network as is encoded in ’boolean_net.m’ and is shown in the Figs. 12,13 and 14. (PDF 110 kb) [file 12885_2018_4756_MOESM1_ESM.pdf]
